# Hospital Sweetness and Light

**Published:** 1893-06-03

**Authors:** Geo. W. Potter


### Hospital Sweetness and Light.

By Geo. W. Potter, M.D.

Professor Huxley has told us in his Romanes Lecture that physiology must yet play its part in the development of life on the broadest possible human lines. Hospital Sunday offers us an occasion on which we may endeavour to show how physiology and medicine touch life and literature at a thousand points. There is little apparent congruity between Arnold the poet and Huxley the physiologist. Nevertheless on the great basis of a common love of truth there is a real consanguinity.

A phrase may describe a world, and by defining may create a new world of knowledge and sympathy in a thinking man's mind. "Sweetness and light" are not ideas ordinarily associated with hospitals; and yet the present writer, ranging many descriptive phrases side by side, unhesitatingly chose this one as best embodying the impression, graved deep upon his consciousness by much experience of the inner life and work of hospitals conducted on modern and scientific lines.

### Sweetness the Watchword.

"Sweetness" is the watchword of modern hospitals above all other words in the language. This single idea has produced one of the greatest of physiological apostles, and tens of thousands of ardent and enthusiastic followers. The sweetness is physical in the first place; but there will not be the slightest difficulty in showing at the proper time that it is mental and moral too. Smells, horrid, pestiferous, sickening, deadly, were characteristic of many hospitals at the beginning, and even at the middle of the present century. The mere effluvia from the half-cleansed bodies of a score or two of poor people in a single ward, and of hundreds of such people in a single building, were often disgusting and poisonous. But when to those natural effluvia were added the dreadful odours of putrefying and mortifying wounds and organs of the body, an atmosphere was produced which carried death to hundreds of patients, and not seldom to nurses and doctors as well. "Hospital fever," "hospital gangrene, "hospitalism," were amongst the most familiar words in the medical vocabulary; and the ideas they connoted were considered then to be as inseparable from hospitals as the soul is from the body.

### An Apostle of Physiology.

Then arose Sir Joseph Lister, the apostle of antiseptics and physical purity. Before Lister's demonstrations the world of putrefaction was a region of chaos and midnight to the average medical intelligence. Why one wound should obstinately "go wrong" and putrefy, whilst another healed in the most obliging manner "by primary union," or, as it was more quaintly expressed, "by first intention," was one of those things which the ordinary medical mind never professed to understand. That the facts were so, and that they were inevitable, were circumstances which apparently could neither be denied nor amended.

The ancient chronicler pictured to himself a world of dark waters and of unbroken night, without order, before reason dawned. He pictured also the spirit of eternal day brooding over the darkness, and proclaiming, "Let there be light!" and light became everywhere diffused, and everywhere beautiful. In a lesser degree, and in tones of beseeching rather than of command, the modern scientific spirit invoked the light of physical knowledge and truth. We see disease, it seemed to say; we see putrefaction and its ten thousand perils and deadly ills. "Let there be light": let us try to master the knowledge of it; let us understand clearly and fully what this putrefaction is and what its cause, in order that by some means we may destroy it, or at least put some check, more or less effectual, upon its intolerable ravages.

### Some Noble Discoverers.

It was in a hospital that the new light dawned which revealed the cause of wound putrefaction, and which, almost simultaneously, made clear the means of prevention and cure. The discovery was like the discovery of a new continent, or of a new art in manufactures. The man who made the discovery, or at any rate, who demonstrated its reality in this country, was our fellow-countryman, Joseph Lister. To us it is all now plain sailing: to him it was pioneer work. He had to grope his way like a half-blind man who sees human beings "as trees walking."

The medical mind cannot help comparing this discovery, of the cause of wound putrefaction, and of the rational means of its prevention or cure, with the discoveries of steam as a motor, of the power-loom and the spinning-jenny, and other discoveries which accelerated the progress of the world a thousand-fold. It may be that each mind is prejudiced in favour of its own things, and cannot judge fairly; but it seems to the present writer that the discovery and demonstration of the part played by living germs in disease processes, and of the means of rendering many of those deadly germs harmless, must properly take rank with the most honourable and valuable discoveries which it has ever fallen to the lot of man to make. We do not yet see this discovery in its true light and proportions. We are too near to it. Moreover, the average medical mind is neither philosophic nor enthusiastic; and so the discoverer is, if not "unhonoured," at least only partially honoured, and altogether "unsung." The philosophic chronicler and historian has yet to arise who shall set in its true proportions the noble achievement of the conservation of life and limb with absolute certainty, by a victory over the minute, and to the naked eye, invisible world, the effect of which must endure to the end of time. It has been given to our contemporary, Sir Joseph Lister, to play a part in the history of human affairs which ranks him with Columbus, Newton, Watt, Stevenson, and all the honoured great ones who have extended the bounds of knowledge and of effectual human conquest over the visible and the semi-invisible worlds. Without a hospital, and the ten thousand circumstances and facilities of a hospital, Lister's great discovery could not, humanly speaking, have been made.

### Only be Clean!

Absolute cleanliness, absolute sweetness in a hospital annihilates at a blow whole worlds of deadly enemies. But did it require many years of effort, ten thousand painful gropings in semi-darkness, and series upon series of practical and unerring demonstrations to convince us of that? Most certainly it did. Surgeons needed to be convinced of the actual existence and power of the deadly "germ" armies before they would take the necessary steps for the fortification of their patients or for the destruction of the besieging microbes. There are some foolish and ignorant surgeons even now who, though taking an infinity of pains in the fortification of their patients by cleanliness against the invasion of germs, yet deny the potency of the germs altogether, and deride the apostle who first showed them the reality of the dangers to be avoided and the means of avoiding them. Stupid creatures these, and ungrateful. They at any rate will make no discoveries, notwithstanding that they are so noisily prompt in kicking down the ladder by which they, as well as others, have risen to competence and notoriety.

It Was All There.

What may be called the "wound microbe" was among the first to be discovered. The horrid smells and putrefactions of the patients' wounds in hospital wards were found to be due to the activity of living forms. These forms, invading wounds by millions, set up suppuration, and gave rise to products which not only poisoned the wound itself, but poured deadly virus into the contiguous parts, and along the streams of blood and lymph into the whole body. Not only so, but exhalations arose from the wounds of the patients, which made the atmosphere of the hospital sickening and pestiferous to all who inhaled it. What, then, was the problem to be solved? The problem was to keep the wound and the germs apart. It was all there! "Not much of a problem," say the unreasoning. Not much to the unthinking, perhaps, any more than America is. But what was the discovery of America to Columbus, to those who sent him out on his adventurous voyages, and to those who first landed with him on the American shores? It was nothing less than a new world. And so to Lister and those who tracked their way with him through the confusing uncertainties of half knowledge and inconclusive experiments, until they set foot upon the solid continent of complete and convincing demonstration, the discovery of the putrefactive and deadly power of germs, and of the means of destroying those germs, or of securing the same end by shutting them out from wounds, was a new world; a new world of restored health and life which became the hope and refuge of every suffering human being for all future time.

The "wound microbe" was but the beginning, the outer fringe of a vast continent of similar knowledge and truth. Hospitals open their doors to tens of thousands of patients who are not suffering from external wounds. Rheumatic fever, pneumonia, typhoid, consumption and a hundred other diseases all supply thousands of victims every year whose deadly maladies were not half understood before the dawn of the microbial pathology, and whose cure was too often a mere lottery from which the unhappy physician or surgeon was doomed to draw out many more blanks than prizes.

The Circling Eddies of Scientific Truth.

The splendid service rendered by the new discovery to hospitals and to the sick in the first instance, is a service to advancing knowledge of the most wide-reaching and universal character. The knowledge of the microbial pathology has given us one clearly-defined principle of universal application in the two regions of prevention and cure. By keeping certain microbes outside man and his wounds we may prevent ten thousand illnesses and diseases, and so may gradually render many of the very hospitals in which this knowledge has been perfected quite superfluous. But, further, by a clear grasp of the nature of the organisms which cause disease, we may go at once to the very root of the matter when a person is attacked by illness, and by destroying the cause check the further progress of the malady, and bring about a cure in half or a third of the time formerly taken to effect it. In other words, if the public will help hospitals and those associated with them to conquer knowledge with more and more completeness, the hospital army will so widely subdue the territory of disease as to make its own partial disbandment a mere question of time.

Now let us consider for a moment the effect of "Hospital sweetness and light" upon the course of human affairs generally in our own epoch. It is manifest that enormous crowds of human beings are massed together in great cities in our times, and tend to mass themselves there more and more. But such massing of millions results in the similar massing of all the waste matter produced by the millions, and in the consequent deadly corruption of the air and soil, unless some means can be found for dealing with the waste so as to render it harmless. How do what we have called "hospital sweetness and light" help us here? They help us to understand clearly the deadly nature of human and animal waste products, and they tell us exactly how to deal with such products in order to render them harmless. In short, the knowledge gained in hospitals, and by hospital physicians, surgeons, and researchers, is the one thing which makes life tolerable, and even possible, in the cities of tremendous populations which are the prodigies of our own time and country. Hospital workers have held up a candle of such illuminating power in this country that the most ignorant of our citizens are now entirely aware of the deadly character of putrefying human waste, and are equally well aware of the antiseptic methods of sweetness which now partially purify the sources of physical life; and which a dangerous crisis forces us to use so extensively and thoroughly that deadly plagues are either checked or prevented altogether.

Cholera Defied.

At the present moment we are all holding our breath in dread apprehension of a cholera invasion. But to what do we look as our first, our second, and our final lines of defence if the plague should actually attack us? We look to antiseptics, to microbicides, to the intelligent isolation of those who are attacked, to the intelligent fortification of those who are not attacked, and to the intelligent destruction of death-dealing microbes by the use of the means which hospital light and sweetness have gradually made us fully conversant with. In a word, although we await the attack of cholera with trembling, as knowing that if it should be made there must be some painful casualties—yet we await it with confidence and firm resolution, knowing that we have the means at disposal, and the intelligence to use them, which will leave us victors on the field. The simplification of our knowledge and the reduction of it to one or two leading principles of absolute definiteness and certainty make us masters of the situation. All this we owe to "hospital sweetness and light."

A Material Buttress of Morals.

But our contention is that the value of physical purity as an annihilator of disease, which hospitals have enabled us to bring to light, is not exhausted when it has played its part in the physical world. The minds of men, searching for the source and authority of moral obligations, are now beginning to turn away somewhat from the investigation of the contents of the human consciousness, and are asking what the great, material, objective, external world has to say upon the subject. Does the solid, substantial world which men touch with their fingers, on which they tread with their feet, upon which they look with their eyes, which gives them the means of life, and compels them to submit to the forces of death—does this material world say anything on the subject of moral obligation? Antisepsis, the potency of physical purity, seems to say that it does. In absolute physical purity and integrity we have a principle which conserves life absolutely, and renders all the deadly microbes which surround us in air and earth and water entirely powerless. Have we anything analogous to this in the region of mind and of moral obligations? This is a question which goes to the roots of social and moral life.

There are principles in morals which have a precisely similar effect upon the moral constitution and life to that produced by absolute physical integrity and purity upon the animal life. Such a principle is the principle of devotion to a Divine person, of loyalty to a religious organization, of obedience to a sense of duty, whether the duty have any other acknowledged sanction than its own innate authoritativeness or not. If it can be shown from the course and history of the external world that physical purity and integrity lead to the noblest physical developments and possibilities, there is an object lesson which may be, which is, of the greatest possible value in the equally human region of morals and moral obligations. If the moral teacher can take his stand upon absolute, demonstrable physical facts and histories, and can point out the all-surpassing value of physical purity and integrity, he is furnished with an Archimedean fulcrum and lever by which he may lift out of the slough of a paralysing and deadening scepticism a critical and unbelieving world. The world hates contradictions in the great and ultimate facts of science and history even more than in the small things of life; and it will not endure them. But the force of analogies, of similarities, and of identities, it cannot and will not deny. Show a strong, reasoning, and independent race of men, like our own for example, that the physical world rewards purity and punishes impurity with absolute sureness and unyielding severity, and they are already disposed to be willingly convinced that the same thing is and must be true in the moral world.

A Generous Overflow.

The forces that make for asepsis, for purity and integrity of all kinds, physical, mental and moral, must at bottom spring from one source. The world is a world of unities, not of contradictions, of order not of chaos. The ranks of these forces of integrity and purity ought to be joined up. There is everything to gain by joining them up. The modern world asks for help in its struggles to believe the true, and to love the beautiful. There is no help so effectual at the present time as the help which the facts and truths and analogies of the material world affords. Show reasoning men that things are certainly thus and thus in the world of the senses, and it is but a short step for them to take in order to see that things must also be thus and thus in the world of moral duty. The sweetness and light of modern hospitals we claim is a sweetness and light which conserve physical health, life, and beauty; but we also contend that this sweetness and light flow over, as sweetness and light always do, beyond the region of the merely physical, and by a simple and beautiful analogy illuminate the regions of moral obligation, and show convincingly that moral purity and moral integrity cannot but produce in the moral sphere the same perfect and mind-conserving results as physical purity in the physical sphere.

Look Deep and See True.

It is not without significance that the greatest religion in the world, the "religion of the reasoning races" as it may be called, had its beginnings in physical and material well-being, and went on to the moral and the spiritual. Every intelligent person will admit that the roots of Christianity are in Judaism. Moses, the great Jew, was a sanitarian and a statesman. He legislated for the physical and material well-being of his tribe. Even the moral obligations he taught the tribe had their most potent sanctions in a divine approval which was to be shown by material rewards. The religion, thus beginning with the body, was completed in the region of mind and morals by Jesus Christ. Thus was the whole man included. We who aim at the highest perfection and happiness of the body are carrying out one part of the propaganda of this religion of the reasoning races; those who aim at the highest perfection of mind and morals are carrying out the other and complemental part of this great and unifying religion. All this means much to the mind which takes the trouble to explore meanings.

The Physiologist and the Priest.

When we ask the Church to help the hospitals we are really asking it to do a part of its own work, to carry out the early and initial principles and practices of its own religion. There are not two religions, the one of the mind and the other of the body, but one, and that the religion of the mind and the body. It is humanity as it now exists which is presented to the reasonable and the good as the object of their self-denying zeal and their brave and intelligent effort: humanity in the persons of the weak and of the wicked; of the laggards in life's struggle; of the feebleminded and the effortless; of the hereditary wrecks and the accidental or the voluntary ruins. These are the disabled whom the strong must carry, stand by, and defend. Professor Huxley teaches us this as well as the Archbishop of Canterbury, Cardinal Vaughan, and the head of the Salvation Army. Of secular and of sacred we know nothing: of Theist and of Atheist we know nothing. What we know is that one half of mankind suffers, and that the other half is strong. The strong half is stronger to-day than it ever was before—stronger in intelligence, stronger in concentration and in sound methods. This strong half, with its one principle of sweetness, wholesomeness, and purity alike in the physical and the moral world, must find no permanent content until the other half, like itself, is as strong, as pure, as intelligent, and as perfect in manhood as it can possibly be made.

